# Application of Vaccination Behavioural Models Based on Modified Proxy Measures for C Scales and Characteristics of Their Determinants in Patients Vaccinated Against Infectious Diseases: A Cross-Sectional Survey

**DOI:** 10.3390/vaccines14080700

**Published:** 2026-08-13

**Authors:** Tomasz Hikawczuk, Dorota Stefanicka-Wojtas, Agnieszka Rusiecka, Maria Kasprzyk-Smardz, Monika Zadka, Norbert Zachara, Jarosław Zachara, Stanisław Karol Manulik, Katarzyna Lomper, Izabella Uchmanowicz, Donata Kurpas

**Affiliations:** 1 Biostatistics Teaching Team, Statistical Analysis Centre, Wroclaw Medical University, 50-368 Wroclaw, Poland; agnieszka.rusiecka@umw.edu.pl; 2 Division of Scientific Research Methodology, Department of Nursing, Faculty of Nursing and Midwifery, Wroclaw Medical University, 51-618 Wroclaw, Poland; dorota.stefanicka-wojtas@umw.edu.pl (D.S.-W.); katarzyna.lomper@umw.edu.pl (K.L.); izabella.uchmanowicz@umw.edu.pl (I.U.); donata.kurpas@umw.edu.pl (D.K.); 3 Medra Kasprzyk-Smardz & Partners, 63-600 Kępno, Poland; medra@kepno.net; 4 Non-Public Healthcare Center “Przychodnia Nowy Dwór”, 54-438 Wroclaw, Poland; monika.zadka@nzoznowydwor.pl; 5 Non-Public Healthcare Center “ReMed”, 32-825 Borzęcin, Poland; norbertzachara@gmail.com (N.Z.); jarzach@gmail.com (J.Z.); 6 Division of Healthcare Organization, Department of Nursing, Faculty of Nursing and Midwifery, Wroclaw Medical University, 51-618 Wroclaw, Poland; stanislaw.manulik@umw.edu.pl

**Keywords:** vaccination behaviour model, vaccination hesitancy, logistic regression analysis, modified C scales, healthcare

## Abstract

**Background**: C scales are important measures that can support the vaccination process by analysing the possible reasons for vaccination hesitancy. In some studies, it is not possible to utilise typical models based on the traditional 3C–7C scales, but it is possible to adjust them by using modified proxy measures and converting individual determinants into variables of similar significance in the logistic regression analysis of a vaccination behavioural model. **Objective**: The aim of this study was to compare the relationship between determinants defining a vaccination behaviour model based on the classic 3C, 5C, and 7C scales with modified proxy measures and two variables (low vaccine-related fear instead of complacency and exposure to misinformation-prone digital sources instead of conspiracy) and the dependent variable, namely, the vaccination status of patients of primary care centres in Poland from 2024 to 2025. **Methods**: A cross-sectional study was conducted using a survey and questionnaire data linked with patient records from three Polish health centres (*N* = 1206, 46.9% rural residents and 53.1% city residents). Demographic data and determinants of three vaccination behaviour models were compared between vaccinated and non-vaccinated patients using Pearson’s χ^2^ test, while behavioural models based on the 3C, 5C, and 7C scales describing the relationship between independent variables and vaccination status (dependent variable) were prepared using logistic regression. Two of seven determinants were changed. **Results**: All determinants used in the three logistic regression behavioural models differed significantly between vaccinated and non-vaccinated patients (*p* < 0.001). Furthermore, independent variables in each behavioural model were significantly related to the dependent variable (*p* < 0.001). With an increasing number of model determinants, Nagelkerke’s *R*^2^ increased from 0.303 to 0.435 and the ROC-AUC from 0.828 to 0.882. However, the VIF value indicated very low multicollinearity of determinants. The Wald test did not reveal a significant relationship between confidence in public health and the dependent variable (*p* > 0.05, *OR* 1.17–0.92 for Models 1 and 3, respectively). **Conclusions**: In the vaccination behavioural models prepared based on empirical data, all determinants were found to be significantly related to the dependent variable. As the number of independent variables in the behavioural model increased, parameters such as the ROC-AUC and Nagelkerke’s *R*^2^ also increased, while the Akaike information criterion, which determines the balance between fitting the model to data and its complexity, decreased. No significant individual relationship was found for the determinant “confidence in public health”, but this result confirms observations from other previous studies describing the impact of pandemic fatigue, misinformation, and conflicting expert information on institutional trust.

## 1. Introduction

Vaccinations are an important element in the protection of individual patients and populations against infectious diseases [1]. At high vaccination rates, pathogens cannot spread within the population, even in the absence of complete vaccination coverage, a phenomenon known as herd immunity or community protection [2]. However, in recent years, the benefits of herd immunity have been diminished by vaccine hesitancy, which involves delaying or refusing vaccination despite vaccine availability [3,4,5,6]. In fact, the WHO [7] defines vaccination hesitancy as one of the ten most important threats to global health. During the COVID-19 pandemic particularly, perceptions of the importance, safety, and effectiveness of vaccines among people aged 18–34 decreased [8]. This intergenerational vaccine gap is a concern among epidemiologists because young parents are a key factor in the development of their children’s immunity [9].

One method introduced in 2012 to examine this problem is the 3Cs vaccination hesitancy model, which considers three basic elements: confidence, defined as trust in the safety and effectiveness of vaccines; complacency, which considers the important of vaccines; and convenience/constraints, which emphasise ease of access [10]. Betsch et al. [11] expanded the model to 5Cs by adding two additional components: calculation, which contains a risk–benefit component related to vaccination; and collective responsibility, which takes into account the individual patient’s interest in vaccination, as well as the responsibility to limit the transmission of the pathogen to close relatives and larger communities. Geiger et al. [12] added two further determinants that affect the vaccination behaviour of patients to the 5Cs model: compliance, which describes support for social monitoring and action to prevent the spread of disease in the population; and conspiracy, which includes conspiracy thinking and belief in fake news. In one case, this model was used in psychometric validation before vaccination among healthcare providers, parents, and adolescents in France [13]. In another, Teichmann et al. [14] empirically compared the use of the 7C scale of vaccination acceptance between Germany and Greece using logistic regression, finding the lowest value of this variable for measles and the highest for COVID-19.

In Poland, data from the Central Statistical Office [15] and the National Health Fund [16] indicate a decline in vaccination rates among the Polish population, especially among children and adults in the first years after the coronavirus wave.

The increase in vaccination hesitancy in recent years has been largely attributed to the politicisation of the entire vaccination process during the COVID-19 pandemic [17]. This process has led to an increase in disinformation about vaccination, which spreads faster than substantive scientific information on the subject [18], in both less economically developed countries and high-income countries, driving increased interest in alternative forms of disease prevention [19]. The new technologies used to produce mRNA vaccines developed during the COVID-19 pandemic, along with the speed of their development, also created the potential for increased vaccination hesitancy due to a lack of reliable knowledge about the subject [20]. Moreover, the emergence of new coronavirus variants reduced the effectiveness of available vaccines, which were not regularly updated to account for new virus mutations [21]. To rebuild patients’ trust in vaccines, it is necessary to provide transparent and accurate information about their effects [22].

The available literature contains a limited number of studies comparing vaccine hesitancy with acceptance of vaccination by patients. Joshi et al. [23] demonstrated greater acceptance of vaccination against COVID-19 among patients who had previously received the influenza vaccine.

Access to healthcare, its quality, and frequency of use by patients are also important factors influencing vaccine acceptance [3]. Huseth-Zosel et al. [24] also concluded that individuals who rely on the healthcare system for information are much more likely to decide to get vaccinated than those who rely on other information sources.

The aim of this study was to compare the explanatory determinants of vaccination behaviour models based on modified proxy measures for the 3C, 5C, and 7C scales in relation to vaccination status among patients who attended primary care centres in Poland from 2024 to 2025.

## 2. Materials and Methods

### 2.1. Participants and Surveys

Data to determine patients’ vaccination acceptance and behaviour in Poland were collected as part of the EUVABECO project between 2024 and 2025 from three health centres located in three different voivodeships (Lower Silesia, Lesser Poland, and Greater Poland). Participants surveyed included adult patients and children (through caregivers) aged 1 to 98 years who were either eligible for or not eligible for vaccination. Those who consented also completed a medical questionnaire regarding vaccination. In total, data were collected from approximately 400 individuals across all centres, yielding *N* = 1206 individual surveys which were included in the demographic analyses in the form of contingency table comparisons, along with verification of the vaccination behavioural models. Demographic data, including sex, age range, place of residence, and religious affiliation, were compared between vaccinated and non-vaccinated behaviour groups using the Pearson χ^2^ test. This type of analysis was also conducted to compare the following independent variables of vaccination behaviour based on the C-scale models applied in the logistic regression analysis: constraints, confidence in public health, low vaccine-related fear, calculation of risk, compliance, and misinformation-prone digital sources. After collection, data were cleaned in digital format to ensure uniformity of recording across variables from each localisation. Data linkage between the surveys and medical questionnaires was considered, with patients in each method sharing the same identity code. After that, independent variables of the vaccination behaviour models were considered using logistic regression, which assessed the relationship of all determinants with the dichotomous variable and each variable individually. Data of patients in the logistic regression model were not included if the respondents provided incomplete answers for at least one independent variable applied in the model (list-wise deletion method).

Vaccination status was applied as a proxy indicator of vaccination acceptance. As this study uses modified proxy measures for the 3C, 5C, and 7C scales rather than the fully validated versions, selected survey items were mapped onto the conceptual domains of these models. Therefore, the findings should be interpreted as model-informed rather than scale-validation results.

### 2.2. Determinants

Three different models of vaccination behaviour against any infectious disease were constructed, and the relationships between independent variables—assessed based on survey responses relating to acceptance of vaccination against any infectious disease—and the dependent variable were determined. The resulting items were mapped onto the modified proxy measures of 3C/5C/7C domains: compliance was changed to low vaccine-related fear; collective responsibility to social influence; and conspiracy to exposure to misinformation-prone digital sources (Table 1).

**Constraints (all three models)**

In this case, patients answered the question “How do you assess the availability of information on vaccinations?”, where 1 indicates “I don’t have an opinion”, 2 indicates “Very weak”, 3 indicates “Weak”, 4 indicates “Medium”, 5 indicates “Good”, and 6 indicates “Very good”.

**Confidence in public health (all three models)**

In this measurement, patients responded on a 6-point scale to the question “How do you assess confidence in the healthcare system?”, where 1 indicates “I don’t have an opinion”, 2 indicates “Very weak”, 3 indicates “Weak”, 4 indicates “Medium”, 5 indicates “Good”, and 6 indicates “Very good”.

**Low vaccine-related fear (all three models)**

In this measure, patients answered the question “Are you not afraid of vaccinations?”, where 0 indicates no answer, 1 indicates “I don’t agree completely”, 2 indicates “I don’t agree”, 3 indicates “Neutral”, 4 indicates “I agree”, and 5 indicates “I agree completely”.

**Calculation of risk (model with five and seven determinants)**

As part of this measurement, six answers to questions in the “What are your main concerns about vaccines?” category were summarised, with each individual answer rated on a scale from 1 to 5, where 1 is “I don’t agree completely”, 2 is “I don’t agree”, 3 is “Neutral”, 4 is “I agree”, and 5 is “I agree completely” (Cronbach’s α = 0.803). The individual variables were as follows: (a) “Possibility of complications or adverse events following immunization”, (b) “Long-term health effects”, (c) “Vaccine composition (e.g., preservatives, adjuvants)”, (d) “Negative impact on the immune system”, (e) “Vaccinations are a pretext for controlling and/or microchipping the population”, and (f) “Vaccinations are driven by pharmaceutical company profits, not public interest”.

**Social influence (model with five and seven determinants)**

In this measure, respondents answered the question “Was the decision to vaccinate associated to any extent by others (family, friends, social groups)?”, where 1 indicates “Never”, 2 indicates “Rarely”, 3 indicates “Sometimes”, 4 indicates “Often”, and 5 indicates “Always”.

**Compliance (model with seven determinants)**

Compliance was considered by analysing answers to the question “Should vaccinations be mandatory?”, where 0 indicates “No”, 1 indicates “I don’t have an opinion”, and 2 indicates “Yes”. It must also be noted that compliance in this case is more focused on the respondent’s attitude towards the mandatory vaccination policy than on willingness to follow the recommendations for the use of sanitary measures.

**Exposure to misinformation-prone digital sources (model with seven determinants)**

**Figure 1:**
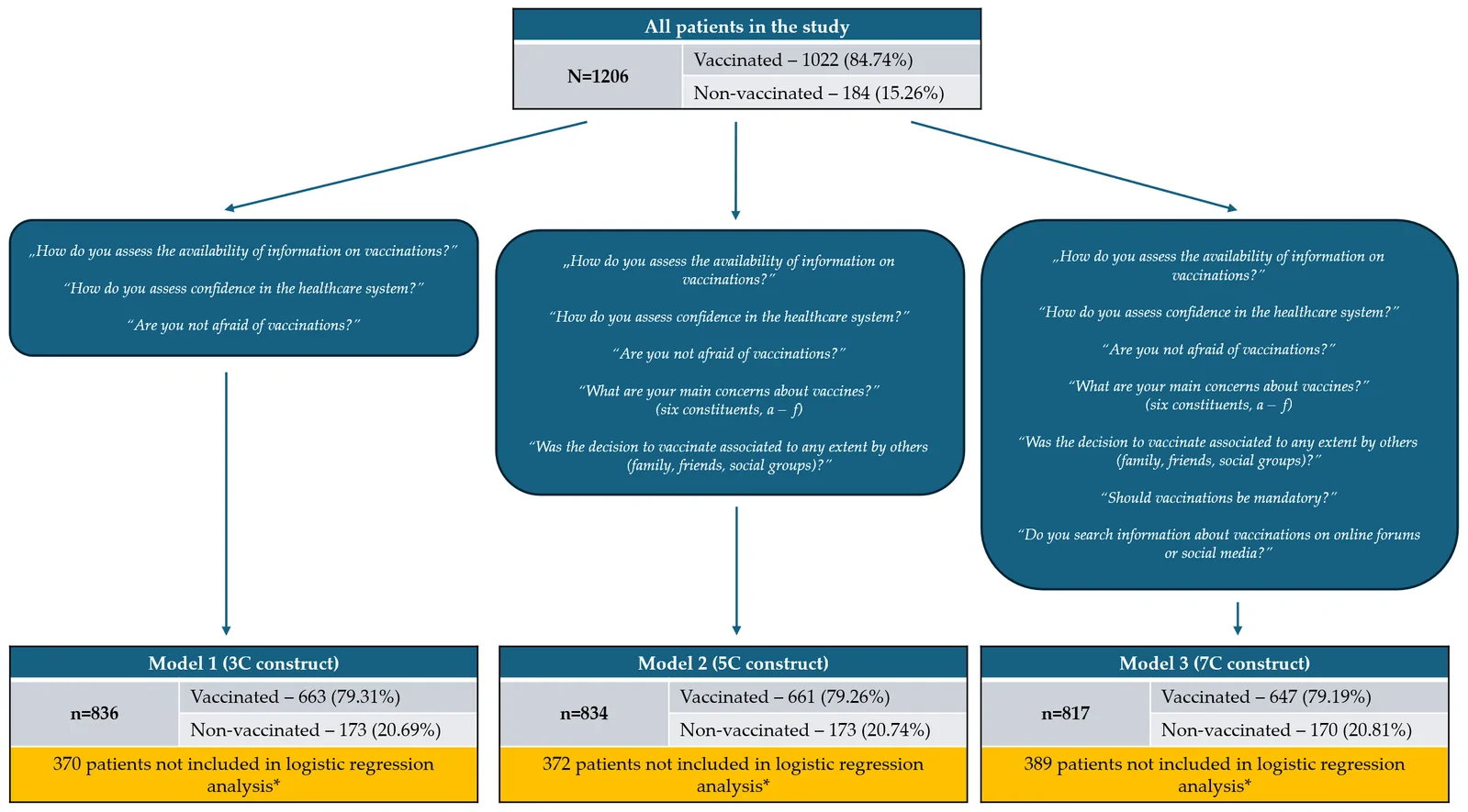
Flow diagram of patients in models using logistic regression. * In logistic regression, the listwise deletion method was used, which resulted in the inability to use patients’ data in analysis if they did not answer one of the questions given in the diagram for each individual model (the number of respondents with missing data was highlighted for each model).

This determinant was based on the question “Do you search information about vaccinations on online forums or social media?”, where 0 indicates “No” and 1 indicates “Yes”. Figure 1 presents the number of vaccinated and non-vaccinated patients in each logistic regression model and overall, considering missing data from respondents.

### 2.3. Statistical Analysis

Statistical analysis was performed using Tibco Statistica 13 (Tibco Software Inc., Palo Alto, CA, USA). Data from patients of various ages (ranging from 1 to 98 years) from three health centres (*N* = 1206) were merged into a single spreadsheet using Office Excel 2021 (Microsoft Inc., Redmont, WA, USA).

Demographic data and determinants of the behavioural models were compared between vaccinated and non-vaccinated patients in the form of contingency tables using Pearson’s χ^2^ test. In the analysis, patients who responded to the survey questions were assessed in terms of demographic variables, and individual independent variables of the behavioural models were considered. Logistic regression analysis was used to describe the models, which determined the association between the independent variables and the assessed dichotomous variable (modelling the acceptance of vaccination against any infectious disease) in a holistic approach with a significance level of *p* < 0.05. Additionally, the significance of the relations of individual components of the C-scale models was also considered using the Wald test at a significance level of *p* < 0.05. The odds ratio (OR) and CI values were also considered to describe individual determinants. If a patient did not answer at least one survey question within the individual independent variables, their data were not included in the logistic regression analysis and model construction (list-wise deletion method).

The following sigmoid equation for logistic regression describing probability was used:

$$p=\frac{e^{z}}{1+e^{z}}$$

where

*z* = *β*_0_ + *β*_1_*x*_1_ + *β*_2_*x*_2_ +…+ *β*_*k*_*x*_*k*_.

The parameters are defined as follows:

*p*—probability;

e—base of natural algorithm;

*β*_0_—intercept;

*β*_1_, *β*_2_, *β_k_*—regression coefficients;

*x*_1_, *x*_2_, *x_k_*—independent variables.

To determine the multicollinearity of the independent variables used, the variance inflation factor (VIF) was used:

$$\mathrm{VIF}\;=\;\frac{1}{1-R_{j}^{2}}$$

where $R_{j}^{2}$ is the coefficient of determination of the model in which the dichotomous variable is explained by the independent variables.

To determine the extent to which the independent variables predict the dichotomous variable, the Nagelkerke *R*^2^ coefficient was determined:

$$R_{NK}^{2}=\frac{R_{CS}^{2}}{1-L_{0}^{\frac{2}{n}}}$$

where

$R_{CS}^{2}$—Cox–Snell *R*^2^;

*L*_0_—credibility of the null model;

*L_M_*—credibility of the null model;

*n*—number of observations.

To compare individual models, the Akaike information criterion (AIC) was applied:

AIC = 2*k* − 2ln (*L*)

where

*k*—number of estimated parameters;

*L*—maximum value of the model function;

ln—natural logarithm.

The Area under the Curve (AUC) value was used to determine the model’s predictive ability to distinguish between acceptance and hesitation or refusal of vaccination:

$$\mathrm{AUC}\;=\;\frac{R_{P}-\frac{n_{P}(n_{P+}1)}{2}}{n_{P}\cdot n_{N}}$$

where

*R_P_*—the sum of ranks for all positive cases;

*n_P_*—total number of positive cases in the database;

*n_N_*—total number of negative cases in the database.

The sensitivity and specificity of the model, describing the model’s ability to correctly recognise acceptance or hesitation/refusal of vaccination, were determined:

$$\mathrm{Sensitivity}\;=\;\frac{TP}{TP+FN}$$

$$\mathrm{Specificity}=\frac{TN}{TN+FP}$$

where

TP—true positives;

TN—true negatives;

FN—false negatives;

FP—false positives.

Accuracy was used to determine the percentage of analysis results that were correctly classified by the model. Precision, on the other hand, was used to determine whether the model was able to predict positive results and to what extent.

$$\mathrm{Accuracy}=\frac{TP+TN}{TP+FP+TN+FN}$$

$$\mathrm{Precision}=\frac{TP}{TP+FP}$$

## 3. Results

### 3.1. Demographic Characteristics

Demographic data were compared using contingency tables for vaccinated and non-vaccinated patients, and observed and expected data were compared using Pearson’s χ^2^ test (Table 2). No statistically significant difference was observed in the case of sex (*p* > 0.05), but statistical differentiation was observed based on age ranges (*p* < 0.05): Among the vaccinated group, the most abundant classes were patients aged 35 to 50, 51 to 65, and 66 and over, all of which exceeded 20.5%; among children, the share was ~13.8%. In the non-vaccinated group, the most abundant class was 35 to 50 years old (41.3%), while the least abundant included children aged 1 to 8 and 9 to 18 years (4.3 and 2.2%, respectively). When analysing place of residence, significant differences were observed (*p* < 0.05): Among vaccinated patients, the most frequent class included patients from rural areas (51.6%), while, in the non-vaccinated group, the most frequent class included patients from cities with more than 200 k citizens (41.8%). In terms of the faith of the patients, the most abundant group was Catholics, accounting for 91.8% of the vaccinated group and 84.2% of the non-vaccinated group.

### 3.2. Qualitative Data as the Main Constituents and Determinants of Behavioural Models

Determinants applied in the behavioural models were also analysed using Pearson’s χ^2^ test in the form of contingency tables (Table S1). All analysed variables were statistically differentiated (*p* < 0.001) when the experimentally observed and expected values were compared. With respect to the constraints’ determinant (“How do you assess the availability of information on vaccinations?”), significant differences were confirmed (*p* < 0.001): The most frequent choices were “Good” and “Medium” (45.6 and 33.7%, respectively), while “I don’t have an opinion” was chosen by 5.2% of patients. In the non-vaccinated group, 32.9% of patients answered “Medium”, and 15.0% chose “I don’t have an opinion”. To assess confidence in public health, the survey question was “How do you assess confidence in the healthcare system?” The most frequent answer in both the vaccinated and non-vaccinated groups was “Medium” (in both cases, 53.2%); the least frequent responses were “I don’t have an opinion” (2.2%) in the vaccinated group and “Very Good” (3.5%) in the non-vaccinated group. When comparing low vaccine-related fear via the question “Do you have any fear of vaccines?”, respondents in the vaccinated group most often chose the “Neutral” answer (33.8%), followed by “I agree” (22.4%) and “I don’t agree” (22.2%); in the non-vaccinated group, patients mainly chose the “Neutral” answer (30.4%), 25.5% had no answer at all, indicating a lack of worries or a factor determining uncertainty or lack of vaccination, and 17.4% chose the “I don’t agree completely” answer.

When analysing calculation of risk and the main concerns about vaccines, the sum of six answers with different constituents was taken into consideration: “Possibility of complications or AEFI” was the first element of this determinant, and a significant difference (*p* < 0.001) was observed, where, in the vaccinated group, the most frequent answers were “I agree” and “Neutral” (50.0 and 35.9%, respectively) and, in the non-vaccinated group, “I agree” and “I agree completely” were emphasised (36.4 and 31.5%, respectively). “Long-term health effects” was the second element of the sum, and, as with the previous constituent, a significant difference was confirmed (*p* < 0.001), where, in the vaccinated group, the most frequent answers were “I agree” and “Neutral” (45.0 and 37.9%, respectively), whereas, in the non-vaccinated group, they were “I agree” and “I agree completely” (35.9% and 29.5%, respectively). A significant relationship (*p* < 0.001) was also observed for the answers relating to “Vaccine composition”: vaccinated patients most frequently chose “Neutral” and “I agree” (51.9 and 26.0%, respectively), while non-vaccinated patients most commonly chose “Neutral” and “I agree” (32.2 and 26.3%, respectively). “Negative impact on the immune system” differed significantly in frequency between vaccinated and non-vaccinated patients: similarly to the answers relating to “Vaccine composition”, the most frequent answers were “Neutral” (51.9 and 32.2% in the vaccinated and non-vaccinated groups, respectively) and “I agree” (26.0 and 26.3%, respectively). Regarding “Vaccinations are a pretext for controlling and/or microchipping the population”, significant differences between groups were observed (*p* < 0.001): in the vaccinated group, 88.6% of patients chose “Neutral”, “I don’t agree”, or “I don’t agree completely”, whereas, in the non-vaccinated group, only 68.6% chose those answers. For “Vaccinations are driven by pharmaceutical company profits, not public interest”, the observed answers differed significantly (*p* < 0.001) from those expected: in the vaccinated group, the most frequent answers were “Neutral” and “I don’t agree” (48.9 and 14.3%, respectively), whereas, in the non-vaccinated group, they were “I agree”, “Neutral”, and “I agree completely” (35.4, 31.0, and 24.3%, respectively).

The social influence determinant (“Was the decision to vaccinate associated to any extent by others (family, friends, social groups)?”) was also significantly differentiated (*p* < 0.001): in the vaccinated group, the most frequent answers were “Sometimes”, “Rarely”, and “Often” (41.6, 21.5, and 18.9%, respectively), while, in the non-vaccinated group, they were “Never”, “Sometimes”, and “Rarely” (33.5, 28.3, and 27.7%, respectively).

With respect to compliance (“Should vaccinations be mandatory?”), significant differentiation was confirmed (*p* < 0.001): in the vaccinated group, the most frequent answer was “Yes” (61.1%), while “No” was less frequent (14.8%), and 24.1% of patients did not express an opinion: in the non-vaccinated group, 43.9% of patients answered “No”, 37.0% said “I don’t have an opinion”, and only 19.1% of respondents answered “Yes”.

The misinformation-prone digital sources determinant (“Do you search information about vaccinations on online forums or social media?”) also showed significant differentiation (*p* < 0.001): in the vaccinated group, 92.6% respondent chose “No”, while, in the non-vaccinated group, this figure was 79.4%.

### 3.3. Models of Vaccination Behaviour

In the analysis, the relationships between all determinants (modified proxy measures) of the vaccination behavioural model and the dependent dichotomic variable were determined (likelihood χ^2^ test), and individual variables were also compared separately using the Wald χ^2^ test (Table 3). Model 1 contained three determinants, Model 2 contained five, and Model 3 contained seven.

For each behavioural model, a significant relationship (*p* < 0.001) between the independent determinants and the dichotomic variable (vaccination acceptance) was confirmed. Nagelkerke’s *R*^2^ value increased with the number of determinants in the logistic regression model (from 0.303 in Model 1 to 0.435 in Model 3); the AIC value decreased with the number of determinants (from 680.27 in Model 1 to 584.74 in Model 3); and the VIF value did not exceed 1.5, indicating very low multicollinearity between the independent variables. The model’s ability to distinguish between vaccination acceptance and non-acceptance among patients based on AUC values increased with the increase in the number of independent variables in the logistic regression analysis, whereas the model’s sensitivity decreased with the number of variables from 0.92 to 0.80 (for three and seven variables, respectively). Specificity (0.63–0.81), accuracy (0.75–0.83), and precision (0.79–0.86) increased with the number of variables in the logistic regression analysis.

When analysing individual determinants after the Wald test in Model 1, significant relationships for constraints (OR = 1.43, CI = 1.21–1.70) and low vaccine-related fear (OR = 3.16, CI = 2.46–4.07) were confirmed (*p* < 0.001). In the case of Model 2, significant (*p* < 0.001) individual relationships were confirmed for constraints (OR = 1.48, CI = 1.24–1.76), low vaccine-related fear (OR = 2.92, CI = 2.23–3.80), social influence (OR = 1.77, CI = 1.43–2.17), and calculation of risk (*p* = 0.007, OR = 0.95, CI = 0.92–0.99). Applying logistic regression analysis to Model 3 confirmed the significant relationships (*p* < 0.001) of constraints (OR = 1.37, CI = 1.14–1.65), low vaccine-related fear (OR = 2.77, CI = 2.10–3.65), social influence (OR = 1.61, CI = 1.30–1.99), and compliance and exposure to misinformation-prone digital sources (*p* = 0.011, OR = 0.46, CI = 0.25–0.84).

## 4. Discussion

Vaccination hesitancy, defined as delayed vaccination or refusal of vaccination despite vaccine availability, is a significant factor influencing the transmission of infectious diseases within populations across countries and continents in the era of globalisation [25]. In Poland in particular, COVID-19 vaccination coverage remains relatively low compared to in several other EU countries [26]; however, Figueiredo et al. [27] noted that the perceived importance of vaccination in Poland increased from 75.6% in 2018 to 80.5% in 2022, while Mirska et al. [28] further indicated considerable regional differences in COVID-19 vaccination coverage in the country, with higher vaccination rates reported in some western and central regions than in several eastern regions.

Differences in attitudes toward vaccination have also been observed internationally [29]. The COVID-19 pandemic substantially influenced public discussion about vaccination, vaccine safety, institutional trust, and health-related misinformation, and concerns surrounding novel vaccine technologies, adverse events, and the need for subsequent vaccine doses were particularly visible during this period [30]. Rzymski et al. [30] noted the willingness of patients with chronic diseases at risk of complications during COVID-19 infection to receive subsequent doses of the vaccine, emphasising the need for scientific discussion and a rationale for their use. At the same time, national data indicated changes in vaccination coverage for other infectious diseases during and following the pandemic period [15,16]. However, these trends are likely a result of multiple factors and should not be attributed solely to misinformation or attitudes toward COVID-19 vaccination. Importantly, vaccine hesitancy is not limited to COVID-19 vaccination; decisions regarding routine childhood immunisation, seasonal influenza vaccination, and other recommended vaccinations may similarly be influenced by perceived disease risk, vaccine safety concerns, access to reliable information, social influences, and trust in healthcare professionals. Thus, although the COVID-19 pandemic provides an important recent context for understanding contemporary vaccination attitudes, our study considers vaccination against infectious diseases more broadly. COVID-19-related evidence is discussed primarily as a relevant contextual example rather than as a direct explanation of the associations observed.

The health centres analysed in this experiment are located in western (Wrocław and Kępno) and southern (Borzęcin) Poland. Previous data have indicated regional differences in vaccination attitudes and COVID-19 vaccination coverage in Poland; however, our analysis is not intended to indicate the superiority of the percentages in different parts of Poland. Rather, it focused on determinants associated with vaccination behaviour that may contribute to identifying groups requiring more targeted vaccination communication and support.

### 4.1. Demographic Data

When analysing data from the three health centres, no significant differences were found in the proportions of women and men among vaccinated and non-vaccinated individuals (*p* > 0.05), but differences were observed across age groups. Notably, the data collected from 1206 patients revealed low percentages of non-vaccinated children in the 1–8 and 9–18 age groups (4.3% and 2.2%, respectively), indicating high vaccination coverage in the analysed health centres. However, for the entire country, it is necessary to consider the entire sociodemographic context.

Take, for example, the study of Hansen et al. [31], which found that the COVID-19 pandemic did not significantly affect childhood vaccination rates in Norway, but vaccination hesitancy remained an obstacle to achieving the recommended influenza vaccination coverage. This illustrates that vaccination behaviour may differ according to both age and the type of vaccine. Indeed, in our study, relatively high proportions of vaccinated participants were observed among individuals aged 51–65 years and ≥66 years, which may partly reflect greater awareness of the potential consequences of infectious diseases among older adults and individuals with comorbidities. This relationship became particularly apparent during the COVID-19 pandemic, when chronic conditions such as diabetes, hypertension, and heart failure were associated with poorer clinical outcomes [32]; nevertheless, similar considerations regarding age, comorbidity, and perceived disease risk are also relevant to vaccination against other infectious diseases, including seasonal influenza.

De Figueiredo et al. [7] emphasised a vaccination gap in the 18–34 age group in their study, which was also observed in our study for the 19–34 age group (16.8% of all non-vaccinated individuals). However, individuals aged 35–50 years constituted the largest proportion of non-vaccinated participants in our sample (41.3%). Kurpas et al. [33] pointed to disinformation about vaccinations on digital and social media as being one possible factor shaping negative attitudes towards vaccinations among parents and guardians/caregivers, which are linked to children’s vaccination rates.

The individual centres used in the analysis comprised a rural health centre, a health centre located in a city of under 50,000 inhabitants, and a health centre in a city of over 200,000 inhabitants. Overall, this resulted in a predominance of people living in rural areas, including patients both at the rural centre and at the centre located in a small city (566 people). The second-largest group included patients from cities with over 200,000 inhabitants (346 people), but a low number of patients from cities with 50,000–200,000 inhabitants was noted (44 people).

Interestingly, the highest percentage of vaccinated individuals among the entire group was recorded among rural residents (51.6% of 1022), while the highest percentage of non-vaccinated individuals was found for patients from cities with over 200,000 inhabitants (41.8% of 184). Ligeti et al. [34], analysing patient data from Hungary, found that vaccination hesitancy was more common among younger adults, those with lower education or financial status, individuals with chronic diseases, and those living outside Budapest. Based on data from Croatia, Bagić et al. [4] reported increased vaccination hesitancy among women, individuals aged 25–34 years, individuals in households with children, residents of smaller settlements, and those with lower levels of education. These findings indicate that the association between place of residence and vaccination behaviour may vary between populations and should be interpreted within the local socioeconomic and healthcare context.

In terms of religion, Hwang et al. [35], in a study including respondents from South Korea, found that younger age, lack of religious affiliation, political conservatism, and low family income were significantly associated with reluctance to get vaccinated. In our study, Catholicism dominated; hence, the highest shares of both vaccinated and non-vaccinated individuals were observed in this class. Among other religions, 6% of non-vaccinated individuals were Orthodox Christians, while 8.2% were those who reported another religion in the survey. However, given the predominance of Catholic participants and the relatively small numbers in some religious categories, these findings should be interpreted cautiously.

### 4.2. Determinants of Vaccination Behavioural Models

Individual determinants of vaccination behaviour, as components of the analysed models, were also statistically analysed using Pearson’s χ^2^ test (Table S1) to check for any differentiation between treatments.

Regarding the constraints determinant and the availability of information with respect to vaccination behaviour, vaccinated patients emphasised the “Good” answer, whereas non-vaccinated participants were less optimistic (“Medium”). This suggests an association between perceived availability of vaccination information and vaccination status. In their publication, Siani and Traner [36] highlighted two related key factors contributing to vaccine distrust and the lack of accurate information: The first was the belief in a conspiracy theory based on the idea that vaccines contain microchips and may be associated with patient decisions or cause infertility. The second was the notion that those vaccines, especially those based on RNA molecules, were approved too quickly. Although some of these concerns became particularly prominent during the COVID-19 pandemic, access to reliable and understandable information is relevant to vaccination decisions more generally.

When analysing the confidence in public health determinant, the most frequent answer chosen by the respondents was “Medium”, but, in the vaccinated group, more optimistic patients were observed, and the second most frequent answer was “Good”, whereas, in the non-vaccinated group, the respondents were more pessimistic (“Weak”). The relatively moderate level of confidence observed in 2024–2025 may partly reflect the broader post-pandemic context, including changes in institutional trust, the politicisation of some public health recommendations, exposure to misinformation, and pandemic fatigue. However, these factors were not directly assessed in the present study and should therefore be considered possible contextual explanations rather than mechanisms demonstrated by our findings. In a study based on data from six European countries, Kyprianidou et al. [32] highlighted that lower trust in government and health authorities, lack of physical distancing, and pandemic fatigue were the main factors associated with patients’ reluctance to get vaccinated. Although these findings concern the specific context of the pandemic, they illustrate the potential importance of institutional trust in vaccination decisions.

Regarding low vaccine-related fear, respondents in the non-vaccinated group were more pessimistic, choosing “Neutral” and more negative answers on the Likert scale, and 25.5% respondents did not answer this question at all. Meanwhile, in the vaccinated group, answers were concentrated around “Neutral”, but patients fluctuated between “I don’t agree” and “I agree”. Lorini et al. [37], specifically in the context of COVID-19 vaccination, emphasised that some individuals who refused vaccination were not opposed to vaccination in general but were instead influenced by concerns about a particular vaccine. Vaccine-related fear should therefore not necessarily be interpreted as general opposition to vaccination and may differ according to the vaccine, disease, and individual perception of risks and benefits.

The calculation of risk focused on five main concerns about vaccines. Responses on a scale of 1–5 were summed for each patient and then included as a determinant value. Additionally, Cronbach’s alpha test was conducted to check the internal consistency of those five variables collectively (α = 0.803). Dziedzic et al. [38] emphasised that protection against individual infection and community transmission and consideration of the potential risks and benefits of vaccination were important factors in vaccination decisions. Differences between vaccinated and non-vaccinated participants were also evident for concerns regarding adverse events following immunisation (AEFI). Indeed, vaccine safety concerns are relevant across vaccination programmes, but this issue received particularly high public attention during the COVID-19 pandemic. Buttery and Clothier [39] highlighted the importance of post-marketing vaccine safety surveillance, because even large Phase III trials may have insufficient statistical power to identify very rare adverse events. The present findings should therefore be interpreted as reflecting broader vaccine safety concerns rather than concerns specific to COVID-19 vaccination. Patients in both groups responded quite similarly to the question about whether vaccine composition significantly affected their vaccination behaviour: neutral responses dominated in both groups, with Likert scale deviations of −1 and +1; however, over 45.2% of vaccinated individuals indicated a neutral response, compared to 33.1% of non-vaccinated individuals. Zaid et al. [25] indicated that the development of new vaccines, the emergence of new variants of infectious diseases, and misinformation about vaccinations appearing on digital platforms are the main factors influencing vaccination hesitancy. When considering the negative impact of vaccinations on the immune system, vaccinated individuals in our study predominantly chose “Neutral”, additionally fluctuating between two adjacent responses on the Likert scale. Meanwhile, the non-vaccinated group preferred “Neutral”, followed by affirmative responses. When determining whether vaccinations are a pretext for population control, vaccinated individuals were characterised by neutral and negative attitudes, while, in the non-vaccinated group, responses ranging from neutral to negative to positive exceeded 30%. These findings suggest that some vaccine-related concerns were more pronounced among non-vaccinated participants; however, the present data do not allow us to determine whether these concerns are the result of insufficient scientific information, exposure to misinformation, previous experiences, or other individual factors.

The COVID-19 pandemic provides a particularly well-documented example of how the information environment may shape vaccination attitudes. Gomes Frugoli et al. [40] emphasised in their study that, in Brazil, the lack of available reliable information on COVID-19 vaccinations was associated with the reluctance of 20% of Brazilians to get vaccinated, while 34% of the country’s residents believed at least one fake news item related to vaccinations posted online. When considering vaccination as a source of profit for pharmaceutical companies, the attitudes of individual groups towards this issue are again evident. Vaccinated individuals chose responses centred around neutral, while, among non-vaccinated individuals, the share of positive responses was close to 60%.

From a social influence perspective, vaccinated individuals most often chose the answer “sometimes” when assessing whether close relatives are associated with their vaccination behaviour, while non-vaccinated individuals chose the answer “never” most often, with nearly 90% of the results falling on a scale of 1–3, indicating lower involvement of close relatives in the vaccination decision. Research by Dziedzic et al. [38] indicated that the most important factors for vaccination among healthcare workers and medical students were health protection (95.2%), family health protection (81.3%), and community health protection (63.3%). Social influences may also be particularly relevant to vaccination decisions involving children and adolescents, where parents or caregivers participate directly in the decision-making process. Nevertheless, the variable used in our study assessed perceived social influence and should not be interpreted as a direct measure of collective responsibility or concern for the health of others.

From the perspective of compliance, in the vaccinated group, 61.1% of patients answered “Yes”, while, in the non-vaccinated group, 43.9% of respondents answered “No” and 37.0% did not have an opinion. Saunders [41] attempted to verify the extent to which vaccinations should be mandatory and suggested a more balanced approach combining obligation with education and promoting public health goals with respect for personal freedom. Instead of the typical mandatory vaccination for all individuals, Saunders proposes an obligation to attend a vaccination centre for a consultation, which, after a substantive discussion, could to some extent persuade those who are particularly hesitant to accept vaccination. It should be emphasised that the compliance proxy used in our study primarily reflected attitudes toward mandatory vaccination and did not comprehensively assess willingness to comply with broader public health recommendations.

The exposure to misinformation-prone digital sources determinant—which takes into account patients’ use of information sources such as online forums and social media, where there is an increased likelihood of finding fake news when searching for information about vaccinations compared to typical medical advice or scientific and medical literature—indicated that the use of such sources was significantly higher among those who decided not to vaccinate (20.6%); in the vaccinated group, this percentage was 7.4%. Taubert et al. [42], in their review of 205 research articles from Europe and North America published in 2012–2022 on conspiracy theories surrounding vaccination against COVID-19, indicated that, depending on social and cultural conditions, the relationship between belief in various conspiracy theories and reduction in vaccination-positive behaviour in individual studies ranged from 2 to 77%. Importantly, however, the variable used in our study measured the use of these information channels rather than actual exposure to misinformation or endorsement of conspiracy beliefs; therefore, the observed association should be interpreted as relating to information-seeking behaviour rather than as direct evidence of conspiracy thinking or the causal effects of misinformation. The relationship between digital information and vaccine hesitancy should also be viewed as a broader vaccination issue rather than one restricted to the COVID-19 pandemic.

### 4.3. Vaccination Behavioural Models

In the conducted analysis, with the application of logistic regression, three models describing vaccination behaviour adapting modified proxy measures and mapping them into classical-form 3C, 5C, and 7C scales were constructed. Despite inclusion of 1206 patients, data from only 817–836 (depending on the model and number of determinants) were analysed; the main reasons for this lower number are the use of a listwise deletion method; the fact that missing data did not coincide with the same persons; and that only persons with complete answers within all individual determinants were taken into account in the analysis. For the calculation of risk determinant, responses from six different risk sources (a–f) were summed, and missing data were included for individuals who did not respond to any of these questions. The numbers of complete data for individual determinants are presented in Table S1.

Missing data in Models 1–3 ranged from 370 to 389 individuals (what respectively represents 30.7 to 32.2% of all patient data). This certainly indicates that attention should be paid to increasing the level of the completeness of surveys in subsequent studies when using this type of method or changing it to one of the multiple implementation methods [43]. However, in this case, we did not want to implement the data. Hence the inclusion of the listwise deletion method and missing data in the logistic regression models.

Analysing Figure 1, the percentages indicate changes of several percent in the proportion of vaccinated and non-vaccinated individuals (up to 6%) in the prepared models and for all patients in the study, which did not significantly change their profile in the individual logistic regression models compared to the total number of patients. The number of uncompleted surveys predominated in the vaccinated group, resulting in a vaccinated to non-vaccinated ratio of approximately 4:1 in the case of data analyses in individual logistic regression models. The number of observations in the individual models is lower due to the use of listwise deletion in the analysis but still exceeds 817 individuals in the individual regression models. This did not affect the risk of loss of external validity and allowed for the demonstration of their internal validity [44].

Table 3 presents the modelling results for the modified proxy measures of vaccination behaviour for three models ranging from three to seven variables. All constructed models indicate that the independent determinants are significantly related (*p* < 0.001) to the dichotomic variable (vaccination decision). In the constructed models, the relations of individual determinants were also compared. Nagelkerke’s *R*^2^ and the ROC-AUC increased with the inclusion of additional behavioural determinants, whereas the AIC was lowest for the most extensive model. VIF values indicated very low multicollinearity among the independent variables; however, because the number of complete observations differed between models, direct comparisons of model performance should be interpreted cautiously.

The given parameters also determine the external and internal validity of the models. Reducing the initial sample size affected the individual types of the model validity. However, all of them contain data from approximately 79% of vaccinated and 21% of non-vaccinated patients and do not indicate extreme disproportions between them, which allows for stable determination of standard errors and odds ratios. In the case of internal validity, Nagelkerke’s *R*^2^ values range from 0.303 to 0.435 (for Model 1 and 3, respectively), indicating a satisfactory and good model fit. Especially for social or medical data in studies not conducted in laboratory conditions (where the values are closer to 1). Additionally, the LR *p*-value in the models indicates a significant effect of the independent variables on the dichotomous variable being studied, and the Wald’s *p*-value allows for determining the significant effect of individual independent variables and accounting for the *OR*. In the case of external validity, specificity ranged from 0.63 to 0.81 (for Model 1 and 3, respectively), indicating that unvaccinated individuals were correctly identified by Model 3 in 81% of cases, while errors occurred in 19% of respondents. The precision value, in turn, ranged from 0.79 to 0.86, and in this respect, Model 3 was also the best, where in the case of vaccinated individuals, the error related to the lower number of respondents compared to the total initial pool of respondents was observed in 14% of cases. Furthermore, the very high external validity of model 3 is also confirmed by the ROC-AUC value of 0.86, indicating a high ability to discriminate between data in terms of the dichotomous variable of the logistic regression model. Analysing all parameters overall, Model 3 is the best, characterized by good internal validity and very good external validity compared to the other two, and the lowest AIC value (it was the most effective in explaining the variability of the dependent variable).

No significant relation was noted in the case of confidence in public health, which could suggest that necessity and awareness of vaccination behaviour are also important aspects in this process. Gomes-Furgoli et al. [40] emphasised that, when defining models of vaccine acceptance (in their case, the 3Cs model), fake news should be limited through greater involvement of healthcare professionals rationally explaining the necessity and mechanisms of vaccination. In our study, general trust in the healthcare system may be insufficient to explain vaccination behaviour in the post-pandemic period. More modified proxy measures such as vaccine-related fear, perceived access to information, social influence, and exposure to digital misinformation could be more precise in the determination of this relationship and may be more relevant for primary care interventions.

In Model 1, constraints and low vaccine-related fear were significantly associated with vaccination status, with higher values being associated with higher odds of being vaccinated. In particular, this latter determinant corresponds with the study of Mongeau et al. [45], which indicated that behavioural determinants known as reasoned action approaches within frameworks of the theory of planned behaviour play a significant role in both the process of getting vaccinated against influenza and vaccine hesitancy among students. The authors emphasised the importance of individual beliefs about the advisability of vaccination, attitudes, and subjective norms influencing vaccination decisions. Importantly, this evidence relating to influenza vaccination also indicates that the behavioural mechanisms considered in the present study are not restricted to the COVID-19 context.

Model 2 revealed significant relationships for four out of five vaccination acceptance modified proxy measures. Low vaccine-related fear, social influence, and constraints were associated with a higher chance of vaccination acceptance, whereas calculation of risk was associated with low vaccination acceptance (5%). Eitze et al. [46] also emphasised that the classical 5Cs model has higher explanatory power than sociodemographic variables.

Models with seven determinants (7Cs) have only been evaluated in a few populations in specific study contexts [31]. In Model 3 in our study, prepared based on data from health centres, five out of seven determinants were individually significantly related to patients’ vaccination behaviour: low vaccine-related fear, compliance, social influence, and constraints were associated with higher odds of being vaccinated, while exposure to misinformation-prone digital sources was associated with lower odds of vaccination. It must also be noted that the compliance determinant primarily considered the attitude of patients towards a mandatory vaccination policy and did not analyse the willingness of given individuals to comply with recommendations and sanitary measures to prevent the spread of infectious diseases.

This third model had the lowest AIC value (584.74) and the highest Nagelkerke coefficient (*R*^2^ = 0.435) among all analysed models, which indicates a very strong model fit to the data. This is consistent with the conclusions of Taubert et al. [42], who, in their review, considered both the results of over 100 correlational studies that indicated a significant negative relationship between conspiracy beliefs and vaccination acceptance and the available literature. Nevertheless, our study did not directly measure conspiracy beliefs, and the association observed for online information sources should not be considered equivalent to associations involving conspiracy thinking.

Overall, the findings should be interpreted within the context in which the study was conducted: data were collected in 2024–2025, shortly after the COVID-19 pandemic, which had a substantial and potentially lasting impact on public discussion regarding vaccination, institutional trust, vaccine safety, and health-related misinformation. COVID-19-related experiences may therefore have formed part of the broader context in which contemporary vaccination attitudes were expressed, and evidence from the pandemic remains relevant to the interpretation of some of our findings. At the same time, the behavioural determinants identified in this study are not necessarily specific to COVID-19 vaccination.

## 5. Practical Implications

The findings of this study have direct relevance for primary care: First, vaccination counselling should not be limited to general reassurance about vaccines. Second, patients with limited perceived access to reliable information may benefit from structured, plain-language educational materials. Third, patients reporting vaccine-related fears require safety-oriented counselling, including transparent discussion of adverse events and long-term concerns. Fourth, patients relying on online forums or social media should be guided toward verified sources and encouraged to discuss doubts with healthcare professionals. Finally, messages emphasising protection of family and community may be particularly useful among patients with moderate hesitancy.

## 6. Limitations

The constructed models were not based on the validated 3C–7C scales but were inspired by them (modified proxy measures); for this reason, they cannot be as precise as standard models.

Since the logistic regression analysis was based on modified versions of the 3C and 7C scales, demographic variables (such as age, place of residence, or religion) significant in the context of comparison in contingency tables were not included as additional components. These variables could have had a significant impact on the resulting model and are a matter worth investigating in future studies.

The public health confidence determinant may not fully reflect its normal impact due to pandemic fatigue after the COVID-19 pandemic and the politicisation of decisions made during this period and could also be associated with misinformation. In the future, it would be worthwhile to consider constructing this determinant based on several components that more accurately describe the impact of public health confidence.

In the conducted study, the vaccination status variable accounts for vaccination against any infectious disease, without differentiating between specific vaccines. This may mask vaccine-specific effects and artificially inflate or deflate the overall strength of individual determinants, which may also be a source of potential bias.

Additionally, the compliance variable and the question “Should vaccinations be mandatory?” were more focused on the attitude of respondents towards the government’s mandatory vaccination policy and thus did not consider the willingness of respondents to follow recommendations and apply appropriate sanitary measures to prevent the spread of infectious diseases.

It should also be mentioned that the exposure to misinformation-prone digital sources determinant analysed only the information search channel and not the cognitive content of beliefs, a determinant which would be much closer to the original intention of conspiracy thinking.

Furthermore, using the listwise method, the number of individual patient data was lower than the number of respondents in the study, which also affected the obtained results and could have led to potential selection bias.

## 7. Conclusions

This multicentre cross-sectional study, conducted in Polish primary care settings, showed that vaccination status was significantly associated with several modified proxy measures derived from the 3Cs, 5Cs, and 7Cs models. Better perceived access to vaccination-related information, lower vaccine-related fear, and greater social influence were consistently associated with higher odds of being vaccinated. Among the compared frameworks, Model 2 appeared to provide a balanced explanatory structure in this dataset, as most of its components were independently associated with vaccination status. However, the optimal model in terms of parameters was Model 3, which added relevant public health insight by showing that support for mandatory vaccination was positively associated with vaccination, whereas seeking vaccine-related information on online forums or social media was negatively associated with vaccination. General confidence in the healthcare system was not an independent predictor in any of the models, which suggests that post-pandemic vaccination strategies in Polish primary care should move beyond general trust-building and focus on concrete, modifiable determinants: accessible information, safety-related counselling, social influence, and reduced exposure to misinformation-prone digital sources.

## Figures and Tables

**Table 1:** Table describing the modified constructs based on the 3C, 5C, and 7C scales.

Item	3C Constructs						
	5C Constructs						
	7C Constructs						
Classical Scales	Constraints	Confidence in Public Health	Compliance	Calculation of Risk	Collective Responsibility	Compliance	Conspiracy
Modified	Constraints	Confidence in Public Health	Low Vaccine-Related Fear	Calculation of Risk	Social Influence	Compliance	Exposure to Misinformation-Prone Digital Sources
Question	“How do you assess the availability of information on vaccinations?”	“How do you assess confidence in the healthcare system?”	“Are you not afraid of vaccinations?”	“What are your main concerns about vaccines?” (six constituents, a–f)	“Was the decision to vaccinate associated to any extent by others (family, friends, social groups)?”	“Should vaccinations be mandatory?”	“Do you search information about vaccinations on online forums or social media?”

**Table 2:** Demographic characteristics of patients.

Item	*N*	Vaccinated	Non-Vaccinated	χ^2^-Value	df	* *p*-Value
	1206	1022	184			
Sex						
Female	775	65.2%	59.2%	2.13	1	0.141
Male	431	34.8%	40.8%			
Age range (years)						
From 1 to 8	149	13.8%	4.3%	64.80	5	<0.001
From 9 to 18	146	13.9%	2.3%			
From 19 to 34	135	10.3%	16.8%			
From 35 to 50	291	21.0%	41.3%			
From 51 to 65	249	20.5%	21.2%			
66 and over	236	20.5%	14.1%			
Place of residence						
Village	566	51.6%	21.2%	58.50	3	<0.001
City < 50 k citizens	250	19.0%	30.4%			
City 50–200 k residents	44	3.1%	6.6%			
City > 200 k residents	346	26.3%	41.8%			
Religion						
Catholicism	1093	91.8%	84.2%	22.19	7	0.002
Other	67	5.1%	8.2%			
Orthodoxy	39	2.7%	6.0%			
Buddhism	2	0.1%	0.5%			
Atheism	2	0.0%	1.1%			
Protestantism	1	0.1%	0.0%			
Jehovah’s Witnesses	1	0.1%	0.0%			
Islam	1	0.1%	0.0%			

* *p*-value statistically significant at *p* < 0.05.

**Table 3:** Components of the modified proxy measures of vaccination behavioural models along with their determinants.

Item	Model 1	Model 2	Model 3
n	836	834	817
**LR *χ*^2^-value**	180.23	216.57	266.89
**LR *p*-value**	<0.001	<0.001	<0.001
***R* ^2^ *_NK_***	0.303	0.358	0.435
**AIC**	680.27	647.01	584.74
**ROC-AUC**	0.828	0.853	0.882
**Sensitivity**	0.92	0.89	0.80
**Specificity**	0.63	0.71	0.81
**Accuracy**	0.75	0.80	0.83
**Precision**	0.79	0.84	0.86
**Coefficient**			
Intercept	−3.532	−3.638	−4.627
Constraints	0.360	0.393	0.320
Confidence in public health	0.156	0.065	−0.085
Low vaccine-related fear	1.151	1.071	1.018
Calculation of risk	-	−0.048	−0.027
Social influence	-	0.569	0.475
Compliance	-	-	0.909
Exposure to misinformation-prone digital sources	-	-	−0.077
**VIF**			
Constraints	1.45	1.46	1.47
Confidence in public health	1.40	1.42	1.44
Low vaccine-related fear	1.08	1.42	1.45
Calculation of risk	-	1.36	1.38
Social influence	-	1.02	1.04
Compliance	-	-	1.16
Exposure to misinformation-prone digital sources	-	-	1.03
**Wald *χ*^2^-value**			
Constraints	17.44	19.27	11.57
Confidence in public health	1.98	0.33	0.49
Low vaccine-related fear	80.59	63.02	51.90
Calculation of risk	-	7.31	1.98
Social influence	-	28.99	18.84
Compliance	-	-	45.03
Exposure to misinformation-prone digital sources	-	-	6.51
**Wald’s *p*-value**			
Intercept	<0.001	<0.001	<0.001
Constraints	<0.001	<0.001	<0.001
Confidence in public health	0.159	0.569	0.485
Low vaccine-related fear	<0.001	<0.001	<0.001
Calculation of risk	-	0.007	0.158
Social influence	-	<0.001	<0.001
Compliance	-	-	<0.001
Exposure to misinformation-prone digital sources	-	-	0.011
***OR***			
Constraints	1.43	1.48	1.37
Confidence in public health	1.17	1.07	0.92
Low vaccine-related fear	3.16	2.92	2.77
Calculation of risk	-	0.95	0.97
Social influence	-	1.77	1.61
Compliance	-	-	2.48
Exposure to misinformation-prone digital sources	-	-	0.46
***CI***			
Constraints	1.21–1.70	1.24–1.76	1.14–1.65
Confidence in public health	0.94–1.45	0.85–1.33	0.72–1.17
Low vaccine-related fear	2.46–4.07	2.23–3.80	2.10–3.65
Calculation of risk	-	0.92–0.99	0.94–1.01
Social influence	-	1.43–2.17	1.30–1.99
Compliance	-	-	1.90–3.24
Exposure to misinformation-prone digital sources	-	-	0.25–0.84

LR or Wald’s *p*-value significant at *p* < 0.05.

## Data Availability

The data presented in this study are available from the corresponding author upon request.

## Supplementary Materials

The following supporting information can be downloaded at https://www.mdpi.com/article/10.3390/vaccines14080700/s1: Table S1: Individual comparison of determinants analysed in experiment.
