# Supplementary material for: Application of Vaccination Behavioural Models Based on Modified Proxy Measures for C Scales and Characteristics of Their Determinants in Patients Vaccinated Against Infectious Diseases: A Cross-Sectional Survey
Source: Vaccines (Basel). 2026 Aug 13;14(8):700. doi: 10.3390/vaccines14080700 (PMC13517135; doi:10.3390/vaccines14080700)
Supplement: Supplementary file 1 [file vaccines-14-00700-s001.zip › vaccines-4426931-supplementary.pdf]

## SUPPLEMENTARY TABLE

**Table S1.** Individual comparison of determinants analysed in experiment.

| Item | *N* | Patient | | $\chi^2$-value | df | **p*-value |
|---|---|---|---|---|---|---|
| | | vaccinated | non-vaccinated | | | |
| | 1206 | 1022 | 184 | | | |
| **Constrains (*n*=946)** | | | | | | |
| I don't have opinion | 66 | 5.2% | 15.0% | 94.4 | 5 | <0.001 |
| Very weak | 25 | 2.5% | 3.5% | | | |
| Weak | 75 | 4.8% | 22.0% | | | |
| Medium | 318 | 33.7% | 32.9% | | | |
| Good | 391 | 45.6% | 22.0% | | | |
| Very Good | 71 | 8.2% | 4.6% | | | |
| **Confidence in Public Health (*n*=947)** | | | | | | |
| I don't have opinion | 29 | 2.2% | 6.9% | 37.7 | 5 | <0.001 |
| Very weak | 39 | 3.6% | 6.4% | | | |
| Weak | 76 | 6.5% | 15.0% | | | |
| Medium | 504 | 53.2% | 53.2% | | | |
| Good | 256 | 29.7% | 15.0% | | | |
| Very Good | 43 | 4.8% | 3.5% | | | |
| **Low vaccine related fear (*n*=848)** | | | | | | |
| Lack of answer | 33 | 0.0% | 25.5% | 290.5 | 5 | <0.001 |
| I don't agree completely | 96 | 10.2% | 17.4% | | | |
| I don't agree | 180 | 22.2% | 15.8% | | | |
| Neutral | 282 | 33.8% | 30.4% | | | |
| I agree | 165 | 22.4% | 3.3% | | | |
| I agree completely | 92 | 11.4% | 7.6% | | | |
| **Calculation of risk (*n*=849)** | | | | | | |
| *Possibility of complications or AEFI (n=797)* | | | | | | |
| I don't agree completely | 17 | 1.6% | 3.0% | 78.4 | 4 | <0.001 |

| | | | | | | |
|---|---|---|---|---|---|---|
| I don't agree | 48 | 5.9% | 5.5% | | | |
| Neutral | 263 | 35.9% | 23.6% | | | |
| I agree | 374 | 50.0% | 36.4% | | | |
| I agree completely | 95 | 6.6% | 31.5% | | | |
| *Long-term health effects (n=771)* | | | | | | |
| I don't agree completely | 19 | 2.1% | 3.9% | 71.4 | 4 | <0.001 |
| I don't agree | 65 | 8.5% | 8.3% | | | |
| Neutral | 268 | 37.9% | 22.4% | | | |
| I agree | 333 | 45.0% | 35.9% | | | |
| I agree completely | 86 | 6.5% | 29.5% | | | |
| *Vaccine composition (e.g. preservatives, adjuvants, n=768)* | | | | | | |
| I don't agree completely | 26 | 3.3% | 4.0% | 70.4 | 4 | <0.001 |
| I don't agree | 110 | 14.6% | 13.2% | | | |
| Neutral | 369 | 51.9% | 32.2% | | | |
| I agree | 200 | 26.0% | 26.3% | | | |
| I agree completely | 63 | 4.2% | 24.3% | | | |
| *Negative impact on the immune system (n=751)* | | | | | | |
| I don't agree completely | 22 | 2.8% | 3.3% | 94.8 | 4 | <0.001 |
| I don't agree | 147 | 23.0% | 6.0% | | | |
| Neutral | 321 | 45.2% | 33.1% | | | |
| I agree | 192 | 24.5% | 29.8% | | | |
| I agree completely | 69 | 4.5% | 27.8% | | | |
| *Vaccinations are a pretext for controlling and/or microchipping the population (n=749)* | | | | | | |
| I don't agree completely | 160 | 21.8% | 19.5% | 41.8 | 4 | <0.001 |
| I don't agree | 193 | 27.7% | 17.5% | | | |
| Neutral | 282 | 39.1% | 31.5% | | | |
| I agree | 74 | 8.1% | 17.5% | | | |
| I agree completely | 40 | 3.3% | 14.0% | | | |
| *Vaccinations are driven by pharmaceutical company profits, not public interest (n=761)* | | | | | | |
| I don't agree completely | 57 | 8.6% | 2.8% | 89.1 | 4 | <0.001 |

| | | | | | | |
|---|---|---|---|---|---|---|
| I don't agree | 139 | 21.1% | 6.3% | | | |
| Neutral | 347 | 48.9% | 31.3% | | | |
| I agree | 139 | 14.3% | 35.4% | | | |
| I agree completely | 79 | 7.1% | 24.3% | | | |
| **Social influence (*n*=940)** | | | | | | |
| Never | 185 | 16.6% | 33.5% | 38.2 | 4 | <0.001 |
| Rarely | 213 | 21.5% | 27.7% | | | |
| Sometimes | 368 | 41.6% | 28.3% | | | |
| Often | 160 | 18.9% | 8.7% | | | |
| Always | 14 | 1.4% | 1.8% | | | |
| **Compliance (*n*=942)** | | | | | | |
| No | 190 | 14.8% | 43.9% | 115.0 | 2 | <0.001 |
| I don’t have an opinion | 249 | 24.1% | 37.0% | | | |
| Yes | 503 | 61.1% | 19.1% | | | |
| **Exposure to disinformation-prone digital sources (*n*=928)** | | | | | | |
| No | 837 | 92.6% | 79.4% | 27.4 | 1 | <0.001 |
| Yes | 91 | 7.4% | 20.6% | | | |

*p-value statistically significant at $p<0.05$.
